# Alternatively activated macrophages at the recipient site improve fat graft retention by promoting angiogenesis and adipogenesis

**DOI:** 10.1111/jcmm.17330

**Published:** 2022-05-16

**Authors:** Ye Li, Xinyao Chen, Lin Liu, Yunzi Chen, Xin Bi, Yuting Chen, Jialiang Zou, Zijue Wang, Ziqing Dong, Feng Lu

**Affiliations:** ^1^ Department of Plastic and Cosmetic Surgery Nanfang Hospital Southern Medical University Guang Zhou China; ^2^ The Plastic and Aesthetic Center The First Affiliated Hospital of Harbin Medical University Harbin China

**Keywords:** fat grafting, inflammation, macrophages, regeneration, vascularization

## Abstract

The inflammatory response mediated by macrophages plays a role in tissue repair. Macrophages preferentially infiltrate the donor site and subsequently, infiltrate the recipient site after fat grafting. This study aimed to trace host‐derived macrophages and to evaluate the effects of macrophage infiltration at the recipient site during the early stage on long‐term fat graft retention. In our novel mouse model, all mice underwent simulated liposuction and were divided into 2 groups. The fat procurement plus grafting (Pro‐Grafting) group was engrafted with prepared fat (0.3 ml). The pro‐Grafting+M2 group was engrafted with prepared fat (0.3 ml) mixed with 1.0 × 10^6^ GFP+M0 macrophages, and then, 2 ng IL‐4 was injected into the grafts on Day 3. In addition, 1.0 × 10^6^ GFP+M0 macrophages were injected into the tail vein for tracing in the Pro‐Grafting group. As a result, GFP+macrophages first infiltrated the donor site and subsequently infiltrated the recipient site in the Pro‐Grafting group. The long‐term retention rate was higher in the Pro‐Grafting+M2 group (52% ± 6.5%) than in the Pro‐Grafting group (40% ± 3.5%). CD34+ and CD31+ areas were observed earlier, and expression of the adipogenic proteins PPAR‐γ, C/EBP and AP2 was higher in the Pro‐Grafting+M2 group than in the Pro‐Grafting group. The host macrophages preferentially infiltrate the donor site, and then, infiltrate the recipient site after fat grafting. At the early stage, an increase in macrophages at the recipient site may promote vascularization and regeneration, and thereby improve the fat graft retention rate.

## 1 INTRODUCTION

Autologous fat transplantation is a method of transferring subcutaneous fat from a donor site to a recipient site.^1^, ^2^ Its major drawback is an unpredictable retention rate.^3^, ^4^ Many studies have focused on the mechanism underlying adipose tissue repair after fat grafting to increase the retention rate.^5^, ^6^

Inflammation occurs in adipose tissue immediately after grafting.^7^ This is mainly mediated by macrophages, which have been detected in transplanted grafts around oil droplets.^8^ M1‐polarized macrophages first appear in the early stages of the inflammatory response and remove free oil and phagocytize dead cells.^5^, ^9^ Then, M1‐polarized macrophages shifts into M2‐polarized macrophages to promote vascularization and regeneration.^10^ Early interference with infiltration of macrophages into fat grafts significantly affects graft retention.^11^ Thus, macrophage, especially M2‐polarized macrophages, infiltration during the early stage is very important for fat graft retention.

Our previous study showed that macrophages were first observed at the donor site and then at the recipient site.^12^ Repair processes and the long‐term retention rate are optimal when adipose tissue is grafted after the donor site no longer requires macrophages for repair.^12^ Thus, we hypothesized that host‐derived macrophages preferentially infiltrate the donor site and subsequently infiltrate the recipient site, and that an increased level of macrophages at the recipient site during the early state improves the fat graft retention rate.

To investigate this hypothesis, we used our novel mouse model, in which simulated liposuction and fat grafting were performed, and traced macrophages. In addition, we investigated the effect of macrophage injection on fat grafting in this mouse model.

## 2 MATERIALS AND METHODS

### 2.1 Animals

All experiments were approved by the Nanfang Hospital Animal Ethics Committee Laboratory and were conducted according to the guidelines of the National Health and Medical Research Council of China. A total of 231 8‐week‐old male C57/BL6 mice and 10 8‐week‐old male GFP mice, obtained from Southern Medical University, were housed in individual cages with a 12 h light/dark cycle and provided standard food and water ad libitum. One hundred and twenty‐six of these mice were anaesthetized by intraperitoneal injection of pentobarbital sodium (50 mg/kg). To prepare fat grafts, bilateral subcutaneous inguinal fat pads were separated and removed from each mouse and placed in culture dishes on ice. Using corneal scissors, these fat pads were gently cut into very small pieces, similar to the size of aspirated fat tissue used clinically. A volume of 0.3 ml of prepared adipose tissue served as the fat graft baseline for each mouse. The graft volume was measured using the liquid overflow method.

### 2.2 Animal model

#### 2.2.1 Pro‐Grafting group and tracing

**FIGURE 1:**
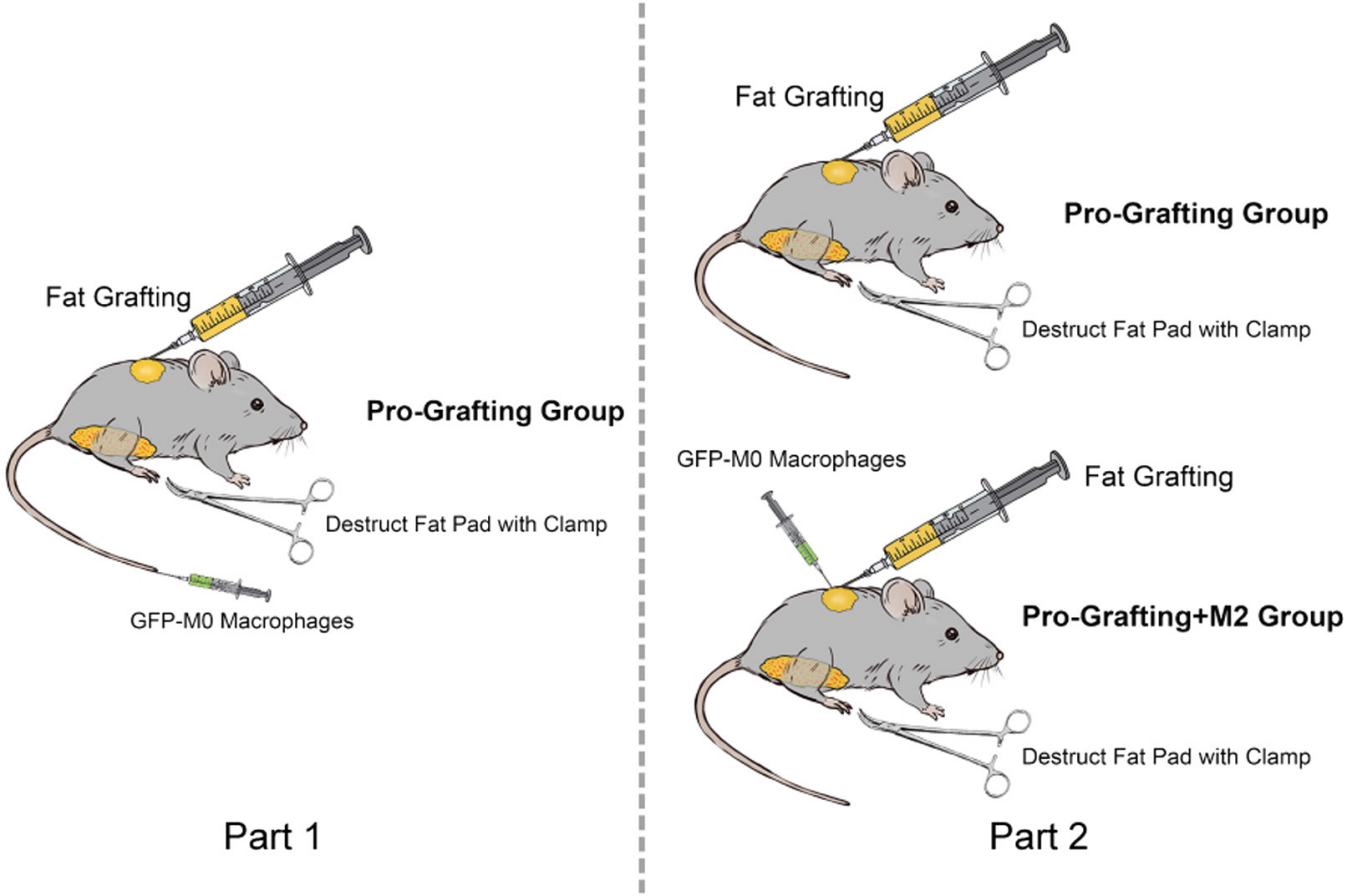
Schematic depiction of the experimental setup

Twenty‐one C57/BL6 mice underwent a simulated liposuction operation by incising the skin of the groin, cutting the subcutaneous fat pad of the groin and crushing it with clips, and then sealing the skin with 7–0 nylon sutures. A 1 ml syringe and an 18‐gauge needle were used to subcutaneously inject 0.3 ml of the prepared fat into each mouse within 30 min after the tissue was harvested. At the same time, 1.0 × 10^6^ GFP+M0 macrophages were injected into the tail vein (Figure 1). The recipient and donor sites were harvested and carefully separated from the surrounding tissues on Days 7, 14 or 30. Each harvested sample was evaluated by immunofluorescence analysis.

#### 2.2.2 Pro‐Grafting and Pro‐Grafting+M2 groups

**FIGURE 2:**
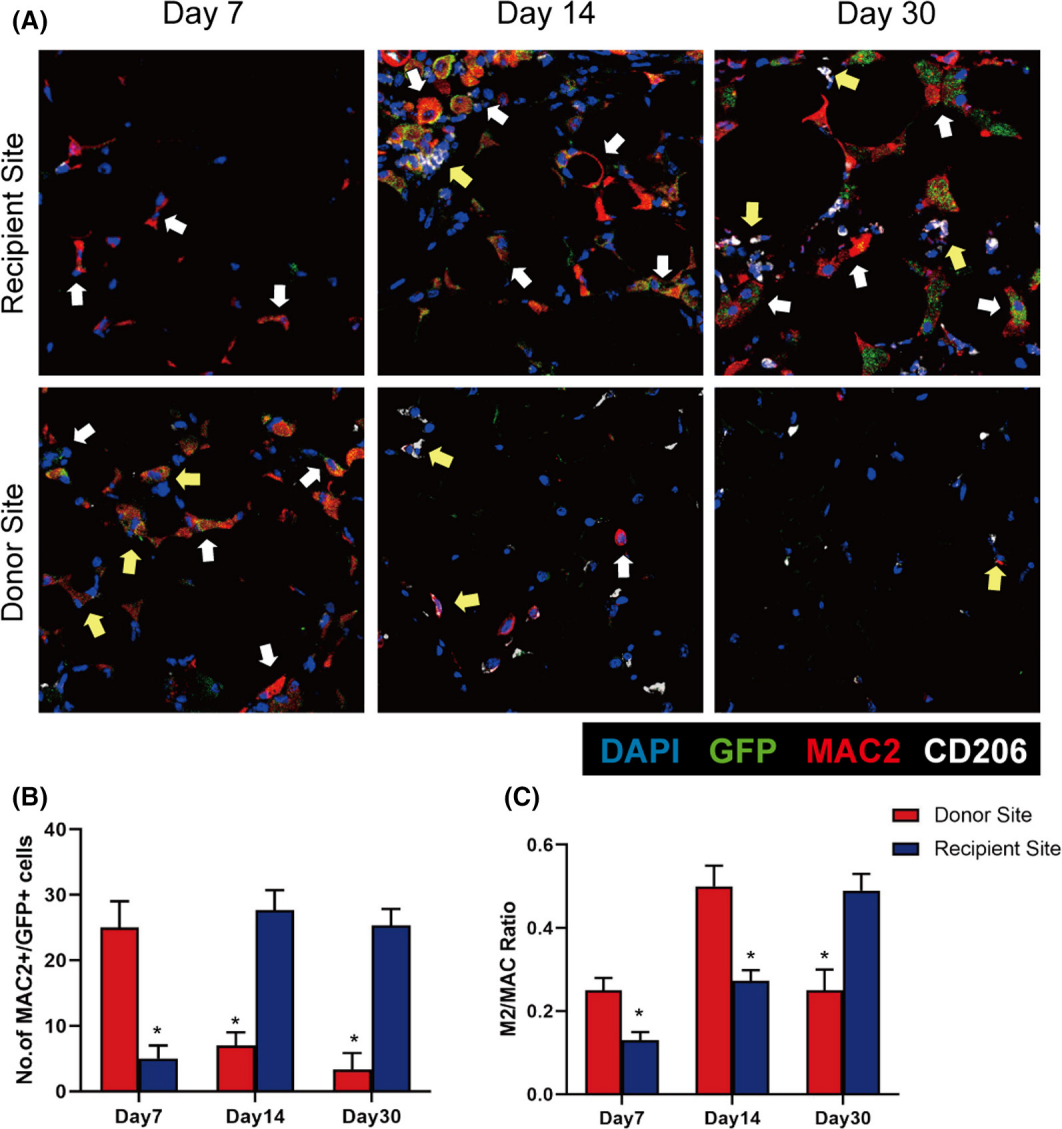
Immunofluorescence staining of recipient and donor sites in the Pro‐Grafting group. (A) M1 macrophages (MAC2+/CD206‐) are indicated by white arrows, and M2 (MAC2+/CD206+) macrophages are indicated by yellow arrows. (B) Quantification of the number of MAC2+/GFP+cells at the recipient and donor sites in the Pro‐Grafting group on Days 7, 14 and 30. (C) Quantification of the M2/MAC ratio at the recipient and donor sites in the Pro‐Grafting group on Days 7, 14 and 30 (right, lower panel). *Donor Site vs. Recipient Site; *p* < 0.05; *n* = 7

Using IBM SPSS version 21.0 (IBM Corp.,), 84 C57/BL6 mice were randomly divided into 2 groups (42 mice per group). The random number table method was used to determine which mice were assigned to which group. Body weight did not significantly differ between the 2 groups. All mice underwent a simulated liposuction operation by incising the skin of the groin, cutting the subcutaneous fat pad of the groin and crushing it with clips, and then sealing the skin with 7–0 nylon sutures. A 1 ml syringe and an 18‐gauge needle were used to subcutaneously inject 0.3 ml of the prepared fat into each mouse within 30 min after the tissue was harvested. In the Pro‐Grafting+M2 group, 1.0 × 10^6^‐prepared GFP+M0 macrophages were immediately injected into grafts (Figure 1) and 2 ng IL‐4, which was used to promote shifting to M2 macrophages, was injected into the grafts on Day 3. As control, 1.0 × 10^6^‐prepared GFP+M0 macrophages and PBS were injected into grafts in the Pro‐Grafting group (Figure 1).

Grafts were harvested and carefully separated from the surrounding tissues on Days 3, 7, 14, 30, 60 or 90 in the 2 groups. Each harvested sample was evaluated by histology, immunofluorescence and Western blot analysis.

### 2.3 Isolation and culture of GFP+M0 macrophages

Brewer thioglycollate is a well‐established sterile agent that increases the migration of monocytes into the peritoneum, allowing the isolation of large numbers of resident, nonmanipulated macrophages. GFP mice were intraperitoneally injected with 2.5 ml of 4% Brewer thioglycollate (Sigma‐Aldrich Chemical Co.,). Four days later, macrophages were collected by peritoneal lavage with 4 ml phosphate‐buffered saline (PBS). Lavage fluid was centrifuged, and cells were plated in Gibco RPMI‐1640 medium (Life Technologies, Inc., Burlington) at a density of 3 × 10^6^ cells per 100 mm polystyrene dish (BD Biosciences, Mississauga,). The macrophage population was allowed to adhere to the plate for 4 h at 37°C in 5% CO2, and then non‐adherent (non‐macrophage) cells were eliminated by aspiration of the supernatant. GFP+M0 macrophages were cultured in RPMI‐1640 medium supplemented with 10% foetal bovine serum (Thermo Fisher Scientific, Inc.,) and 10 mg/ml Gibco penicillin/streptomycin.

### 2.4 Histological examination

Tissue samples were fixed in 4% paraformaldehyde, dehydrated and embedded in paraffin for haematoxylin‐eosin staining. Tissue blocks were sectioned, examined under an Olympus BX51 microscope and photographed using an Olympus DP71 digital camera.

### 2.5 Immunohistochemistry

Tissue samples were stained with rat anti‐mouse MAC2 (1:200; Cedarlane Corp., Burlington,), rabbit anti‐mouse CD206 (1:300; Abcam,), rat anti‐CD31 (1:200, Abcam), rat anti‐CD34 (1:200, Abcam), rabbit anti‐mouse perilipin (1:400; Progen, Heidelberg, Germany) and chicken anti‐GFP (1:200, Abcam) primary antibodies. After washing, the samples were incubated with donkey anti‐rat Alexa Fluor 555 IgG (Abcam) and goat anti‐rabbit Alexa Fluor 430 IgG (Invitrogen, ) antibodies. Nuclei were stained with DAPI (Sigma, ).

### 2.6 Western blotting

Total cell lysates were prepared from adipose samples using M‐PER Mammalian Protein Extraction Reagent (Thermo Fisher Scientific, Inc.). A bicinchoninic acid protein assay (Thermo Fisher Scientific, Inc.) was used to estimate the protein concentration. After separation by sodium dodecyl sulphate polyacrylamide gel electrophoresis using a NuPAGE electrophoresis system, protein extracts were transferred to immobilon polyvinylidene difluoride membranes (Millipore,). Membranes were blocked in 5% milk and then incubated with anti‐PPAR‐γ (1:200, Abcam), anti‐C/EBP (1:1000, Abcam) and anti‐AP2 (1:1000, Abcam) primary antibodies. Thereafter, membranes were incubated with following secondary antibodies: horse‐radish peroxidase (HRP) conjugated Goat Anti‐Mouse IgG (1:5,000; Wuhan Boster Biological Technology) and HRP‐conjugated Goat AntiRabbit IgG (1:5,000; Wuhan Boster Biological Technology). A WesternBreeze Chemiluminescent Detection Kit (Thermo Fisher Scientific, Inc.) was used to detect signals. GAPDH served as an internal control.

### 2.7 Statistical analysis

Data are expressed as mean ± standard deviation and were analysed by a repeated measures analysis of variance. The independent Student's *t*‐test was used to compare the groups at a single time point, while a one‐way analysis of variance was used to compare the groups at all time points. A *p*‐value <0.05 was considered statistically significant.

## 3 RESULTS

### 3.1 Macrophage infiltration at the donor and recipient sites in the Pro‐Grafting group

Immunofluorescence analysis detected many MAC2+/GFP+macrophages (white and yellow arrows), some of which were MAC2+/CD206+/GFP+M2 macrophages (yellow arrows), at the donor site on Day 7 in the Pro‐Grafting group. Although many MAC2+/GFP+macrophages (white and yellow arrows) were observed at the recipient site on Day 14, it took until Day 30 for the level of MAC2+/CD206+/GFP+M2 macrophages at the recipient site to be similar to that at the donor site on Day 7 in the Pro‐Grafting group  (Figure 2A).

Quantitative analysis showed that the number of MAC2+/GFP+macrophages was higher at the donor site than at the recipient site on Day 7 and vice versa on Days 14 and 30 (Figure 2B) (*p* < 0.05). The number of MAC2+/GFP+macrophages at the recipient site remained high until Day 30 (Figure 2B). The ratio of M2/macrophages peaked on Day 14 at the donor site and on Day 30 at the recipient site respectively (Figure 2C).

### 3.2 Comparison of fat graft retention between the 2 groups

**FIGURE 3:**
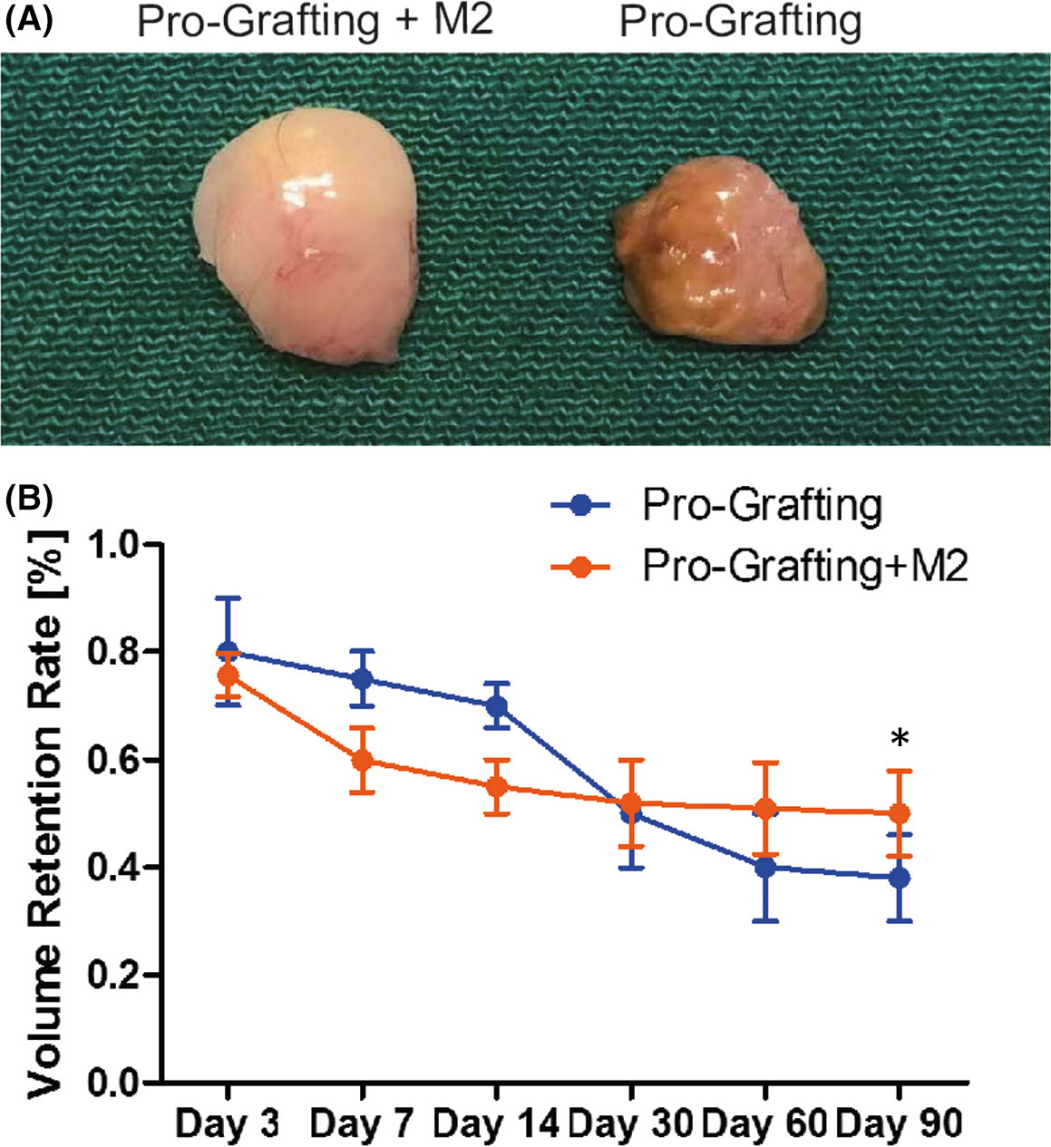
(A) Appearance of grafts in the Pro‐Grafting+M2 and Pro‐Grafting groups on Day 90. (B) The volume retention rate of grafts in the Pro‐Grafting+M2 and Pro‐Grafting groups over time. *Pro‐Grafting+M2 vs. Pro‐Grafting; *p* < 0.05; *n* = 7

The texture of grafts was better in the Pro‐Grafting+M2 group than in the Pro‐Grafting group on Day 90 (Figure 3A). Quantification of graft volume indicated that the adipose tissue volume decreased sharply up to Day 7 in the Pro‐Grafting+M2 group. The rate of decline subsequently decreased, with the adipose tissue volume remaining stable from Day 60 to Day 90. By contrast, the adipose tissue volume in the Pro‐Grafting group decreased slowly from Day 3 to Day 7 and more sharply from Day 7 to Day 30 and remained constant thereafter. The fat graft retention rate at Day 90 was significantly higher in the Pro‐Grafting+M2 group (52% ± 6.5%) than in the Pro‐Grafting group (40% ± 3.5%) (Figure 3B).

### 3.3 Comparison of macrophage infiltration between the 2 groups

Immunofluorescence analysis detected many MAC2+/GFP+macrophages (white and yellow arrows), some of which were MAC2+/CD206+/GFP+M2 macrophages (yellow arrows), on Days 3 and 7 in the Pro‐Grafting+M2 group (Figure S1, lower panel). Many MAC2+ macrophages (white and yellow arrows) were observed on Day 14, and some had become MAC2+/CD206+ M2 macrophages (yellow arrows) on Day 30 in the Pro‐Grafting group (Figure S1, upper panel).

**FIGURE 4:**
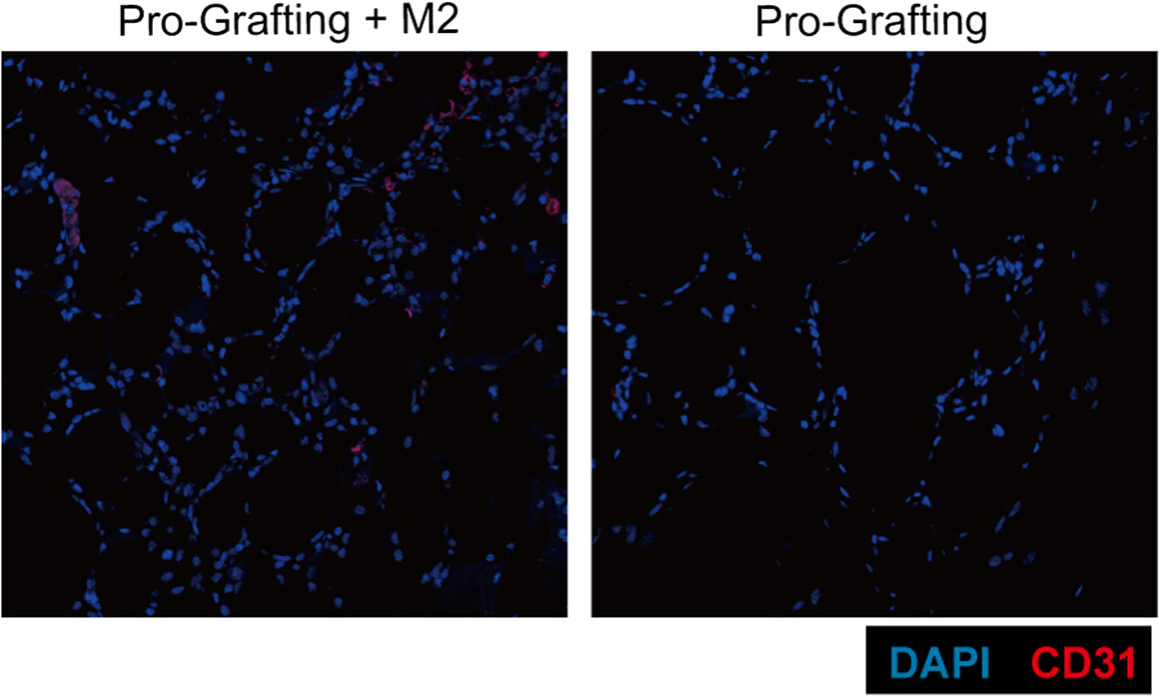
Immunofluorescence staining of grafts for CD31 in the Pro‐Grafting and Pro‐Grafting+M2 groups on Day 14

**FIGURE 5:**
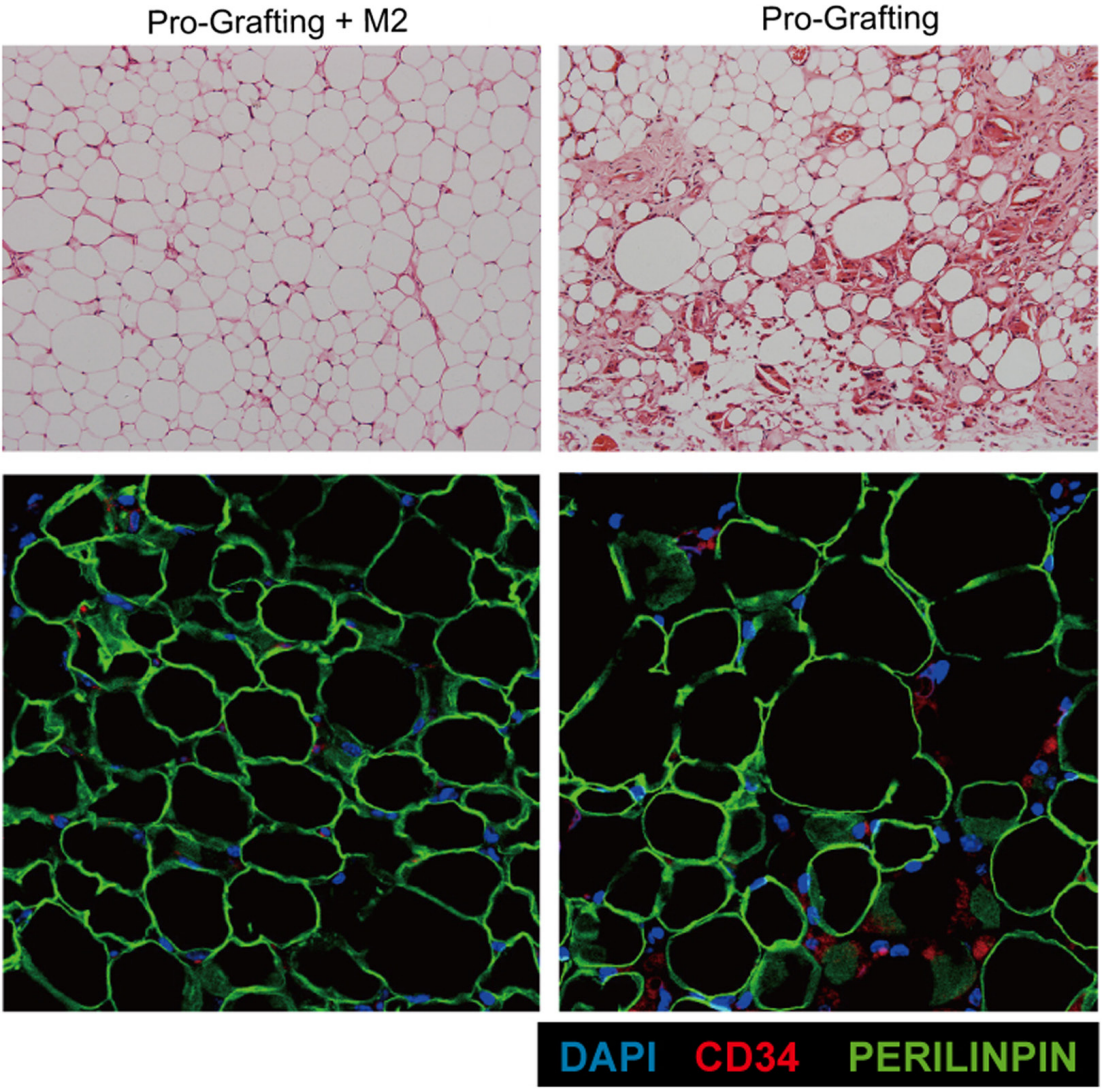
Haematoxylin‐eosin staining of grafts in the Pro‐Grafting and Pro‐Grafting+M2 groups on Day 90 (upper panel). Immunofluorescence staining of grafts in the Pro‐Grafting and Pro‐Grafting+M2 groups on Day 90 (lower panel). DAPI (blue) indicates nuclei. CD34 (red) indicates stem cells. Perilipin (green) indicates adipose cells

**FIGURE 6:**
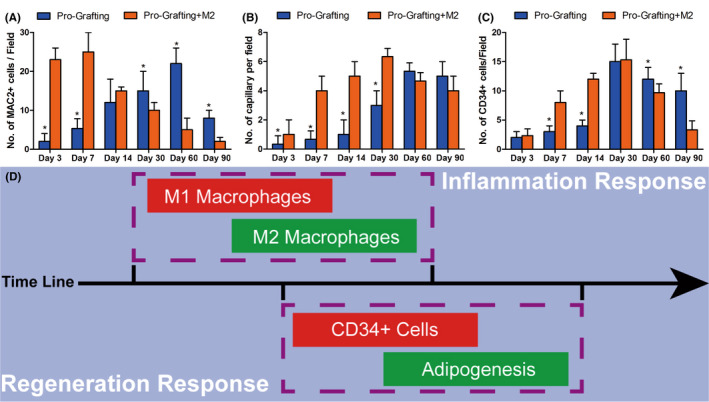
Quantification of the numbers of (A) MAC2+ macrophages, (B) capillaries and (C) CD34+ cells per field in the Pro‐Grafting and Pro‐Grafting+M2 groups over time. (D) Schematic depiction of the inflammatory and regenerative responses after fat grafting. M1 macrophages initiate the inflammatory response, followed by infiltration of M2 macrophages, which recruit CD34+ cells to initiate the regenerative response in fat grafts. *Pro‐Grafting+M2 vs. Pro‐Grafting; *p* < 0.05; *n* = 7

Quantitative analysis showed that the number of MAC2+ macrophages per field was higher in the Pro‐Grafting+M2 group than in the Pro‐Grafting group on Days 3 and 7 and vice versa on Days 30, 60 and 90 (Figure 6A) (*p* < 0.05).

### 3.4 Comparison of vascularization between the 2 groups

Immunofluorescence analysis detected more CD31+ areas in the Pro‐Grafting+M2 group than in the Pro‐Grafting group on Day 14 (Figure 4). Quantitative analysis showed that the number of capillaries per field was higher in the Pro‐Grafting+M2 group than in the Pro‐Grafting group on Days 3, 7, 14 and 30 (Figure 6B) (*p* < 0.05).

### 3.5 Comparison of adipogenic protein expression between the 2 groups

Expression of the adipogenic proteins PPAR‐γ, C/EBP and AP2 on Day 30 was higher in the Pro‐Grafting+M2 group than in the Pro‐Grafting group (Figure S2).

### 3.6 Comparison of regeneration between the 2 groups

Histological analysis showed that the structure of grafts was better in the Pro‐Grafting+M2 group than in the Pro‐Grafting group on Day 90. The level of fibrosis was higher in the Pro‐Grafting group than in the Pro‐Grafting+M2 group on Day 90 (Figure 5, upper panel). Immunofluorescence analysis also showed that the structure of grafts was better in the Pro‐Grafting+M2 group than in the Pro‐Grafting group on Day 90. More CD34+ cells were observed in the Pro‐Grafting group than in the Pro‐Grafting+M2 group on Day 90 (Figure 5, lower panel).

Quantitative analysis showed that the number of CD34+ cells per field was higher in the Pro‐Grafting+M2 group than in the Pro‐Grafting group on Days 7 and 14 and vice versa on Days 60 and 90 (Figure 6C) (*p* < 0.05).

After fat grafting, the inflammatory response, which involves infiltration of M1 and M2 macrophages, is initiated in adipose tissue, followed by the regenerative response, which involves infiltration of CD34+ cells and adipogenesis (Figure 6D).

## 4 DISCUSSION

This study showed that the host‐derived macrophages preferentially infiltrate the donor site rather than the recipient site during early period after fat grafting, and that an increased level of macrophages at the recipient site improves the fat graft retention rate.

The number of host‐derived macrophages are limited, which leads to insufficient local macrophage infiltration when more or larger areas need to be repaired after fat grafting.^13^, ^14^, ^15^ Since the donor site can induce the infiltration of most host‐derived macrophages, the number of macrophage infiltration at the recipient site is not enough to initiate an inflammatory response.^12^ A similar finding was made in this study by cell tracing. Macrophages can be recruited by inflammatory factors, including IL‐1β, IL‐6 and TNF‐α.^16^, ^17^, ^18^ Our previous study demonstrated that the levels of IL‐1β, IL‐6 and TNF‐α are higher at donor sites than at recipient sites after fat grafting, which may cause the different infiltration of macrophages.^12^ Thus, we suggest that donor sites immediately release IL‐1β, IL‐6 and TNF‐α into the blood and recruit host‐derived macrophages, resulting in a lower level of macrophages at recipient sites during the inflammatory stage after fat grafting.

Macrophages have been reported to play a significant role in tissue inflammation and regeneration.^11^, ^12^, ^19^ In the early stage of the inflammatory response, high levels of inflammatory factors induce M1 macrophages polarization and initiate the inflammatory response.^20^ Then, M2 macrophages polarization and produces anti‐inflammatory factors to solve the inflammatory response and promote tissue repair.^21^, ^22^ M2 can promote vascularization and tissue regeneration,^11^, ^23^ but delayed infiltration of M2 may result in poorer vascularization^24^ and long‐term infiltration of M2 can also promote tissue fibrosis.^25^ Thus, matched infiltrating macrophages during the inflammatory stage are important for tissue vascularization and regeneration after grafting.^11^, ^12^, ^13^

In this study, we observed that infiltration of macrophages was delayed at recipient sites; thus, we used macrophages to assist fat grafting in the mouse model. Vascularization, regeneration and consequently, the long‐term retention rate were increased in the Pro‐Grafting+M2 group. For vascularization, M0 macrophages rapidly become M1 macrophages after injection, which may initiate the inflammatory response as soon as possible.^26^ IL‐4 injection can promote M2 macrophage shifting on Day 3.^27^ M1 and M2 macrophages provide a sustained and autologous source of proangiogenic factors such as VEGF and bFGF.^28^ If present in sufficient numbers, macrophages can continuously release angiogenic factors and promote vascularization during the inflammatory stage after grafting, which can provide a good microenvironment and sufficient seed cells for the subsequent regenerative stage.^9^, ^11^ Adipose‐derived stem cells (ASCs), which were detected by the expression of CD34,^6^, ^29^ rapidly and extensively infiltrated during the regenerative stage in the Pro‐Grafting+M2 group. For regeneration, M2 macrophages can drive pluripotent ASCs within fat grafts to proliferate and differentiate into mature lipid‐laden adipocytes by producing soluble factors.^30^ Sequential expression of PPAR‐γ, C/EBP and AP2 plays an important role in terminal differentiation of preadipocytes into triglyceride‐laden adipocytes.^31^ Expression of these proteins was higher in the Pro‐Grafting+M2 group than in the Pro‐Grafting group. Vascularization and regeneration were promoted, and consequently, the long‐term retention rate was increased in the Pro‐Grafting+M2 group.

The retention rate decreased rapidly in the first 7 days in the Pro‐Grafting+M2 group, while it did not begin to decrease rapidly until Day 14 in the Pro‐Grafting group. The number of macrophages began to decrease from Day 7 in the Pro‐Grafting+M2 group and began to increase from Day 14 in the Pro‐Grafting group. Therefore, we hypothesize that the retention rate is closely related to macrophage infiltration during the early stage after fat grafting.^32^ After grafting, macrophages must rapidly infiltrate and then withdraw from adipose tissue, which may increase the retention rate.^12^ If infiltrated macrophages remain for a long time, fibrosis may increase, and the long‐term retention rate may decrease^13^, ^32^. In summary, rapid infiltration and subsequent withdrawal of macrophages during the early stage after fat grafting are required to increase the long‐term retention rate.

In clinical situations, injury of the donor site before fat grafting is unavoidable, and consequently, macrophage infiltration at the recipient site might be insufficient after fat grafting. Based on this study, we propose the following clinical recommendations. First, injury at the donor site should be minimized for example, by reducing the area of liposuction when sufficient fat has been collected for grafting. Second, macrophages should be used to assist fat grafting at the recipient site. Third, drugs should be used to induce macrophage infiltration at the recipient site or inhibit macrophage infiltration at the donor site. Further clinical trials and animal experiments are needed.

## 5 CONCLUSIONS

Host‐derived macrophages preferentially infiltrate the donor site and subsequently infiltrate the recipient site after fat grafting. During the early stage, an increased level of macrophages at the recipient site promotes vascularization and regeneration and thereby increases the fat graft retention rate.

## AUTHOR CONTRIBUTION

**Ye Li:** Conceptualization (equal); Data curation (equal); Formal analysis (equal); Writing – original draft (lead). **Xinyao Chen:** Conceptualization (equal); Data curation (equal); Writing – original draft (equal). **Lin Liu:** Methodology (equal); Resources (equal). **Yunzi Chen:** Data curation (equal); Formal analysis (equal); Methodology (equal). **Xin Bi:** Data curation (equal); Formal analysis (equal). **Yuting Chen:** Methodology (equal); Resources (equal). **Jialiang Zou:** Data curation (equal); Formal analysis (equal). **Zijue Wang:** Conceptualization (equal); Data curation (equal); Methodology (equal). **Ziqing Dong:** Conceptualization (equal); Funding acquisition (equal); Project administration (equal); Writing – review & editing (equal). **Feng Lu:** Conceptualization (equal); Funding acquisition (equal); Methodology (equal); Project administration (equal); Writing – review & editing (lead).

## CONFLICT OF INTEREST

The authors declare that they have no conflict of interest.

## Supporting information

Fig S1

Fig S2

Supplementary Material

## Data Availability

The data sets used and/or analysed during the current study are available from the corresponding author on reasonable request.
