# Supplementary figures and images for: Alternatively activated macrophages at the recipient site improve fat graft retention by promoting angiogenesis and adipogenesis
Source: J Cell Mol Med. 2022 May 16;26(11):3235–42. doi: 10.1111/jcmm.17330 (PMC9170812; doi:10.1111/jcmm.17330)

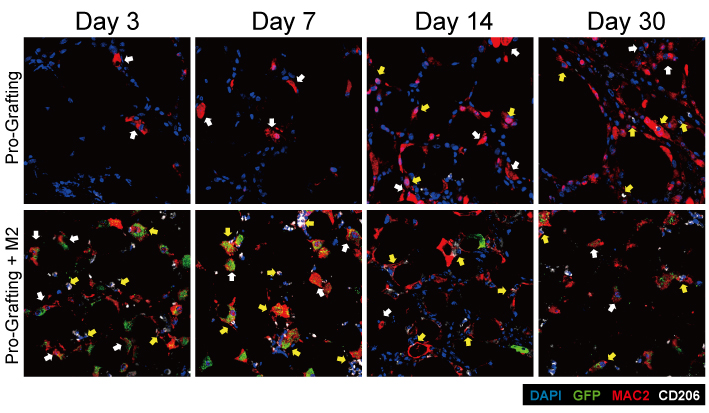

Supplement: Supplementary file 1 — Fig S1 [file JCMM-26-3235-s003.jpg]

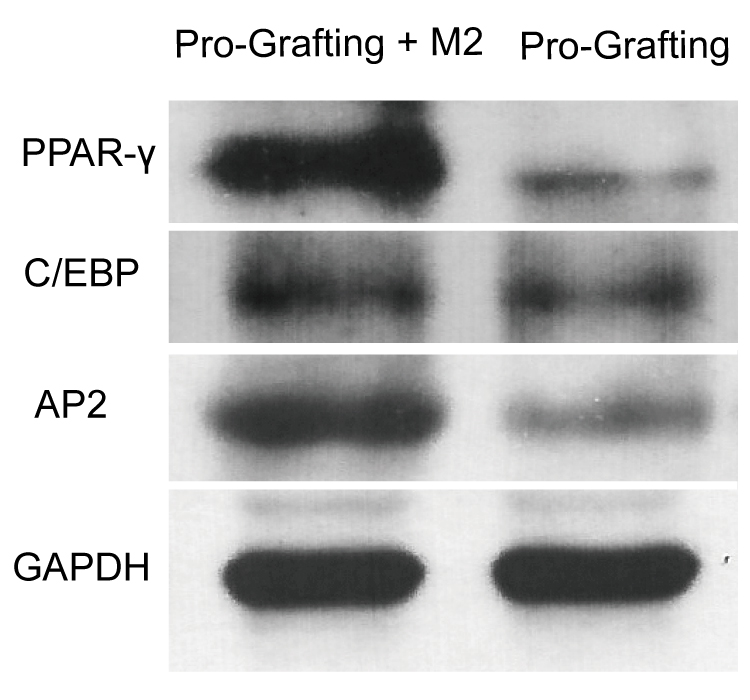

Supplement: Supplementary file 2 — Fig S2 [file JCMM-26-3235-s001.jpg]
