# Supplementary material for: Alternatively activated macrophages at the recipient site improve fat graft retention by promoting angiogenesis and adipogenesis
Source: J Cell Mol Med. 2022 May 16;26(11):3235–42. doi: 10.1111/jcmm.17330 (PMC9170812; doi:10.1111/jcmm.17330)
Supplement: Supplementary file 3 — Supplementary Material [file JCMM-26-3235-s002.docx]

Figure S1 Immunofluorescence staining of grafts in the Pro-Grafting and Pro-Grafting+M2 groups on Days 3, 7, 14 and 30. MAC2 (red) indicates macrophages. M1 macrophages were defined as MAC2+/CD206-cells (white arrows). M2 macrophages were defined as MAC2+/CD206+ cells (yellow arrows).

Figure S2 Western blot analysis of PPAR-γ, C/EBP and AP2 in grafts in the Pro-Grafting+M2 and Pro-Grafting groups on Day 30.
